# Supplementary material for: Characterization and Preliminary Application of a Novel Lytic Vibrio parahaemolyticus Bacteriophage vB_VpaP_SJSY21
Source: Int J Mol Sci. 2023 Dec 15;24(24):17529. doi: 10.3390/ijms242417529 (PMC10744069; doi:10.3390/ijms242417529)
Supplement: Supplementary file 1 [file ijms-24-17529-s001.zip › ijms-2717552-supplementary.pdf]

Table S1. Functional annotation of bacteriophage genome NR database protein.

Number	Start (bp)	End (bp)	Size (aa)	Target Number	Prediction of Protein Functions
ORF 1	1	1686	561	AUG88511.1	DNA polymerase
ORF 3	1892	2065	57	QIG72566.1	Hypothetical protein
ORF 5	2243	2482	79	AUG88365.1	Hypothetical protein
ORF 6	2472	2651	59	AUG88366.1	Hypothetical protein
ORF 7	2633	3022	129	AUG88367.1	Hypothetical protein
ORF 8	3023	3328	101	AUG88368.1	Hypothetical protein
ORF 9	3321	3497	58	AUG88369.1	Hypothetical protein
ORF 10	3487	3651	54	AUG88370.1	Hypothetical protein
ORF 12	3964	4173	69	AUG88372.1	Hypothetical protein
ORF 13	4176	4568	130	AUG88373.1	Hypothetical protein
ORF 14	4562	4726	54	AUG88374.1	Hypothetical protein
ORF 15	4716	4916	66	AUG88375.1	Hypothetical protein
ORF 16	4974	5279	101	AUG88376.1	Hypothetical protein
ORF 17	5291	6040	249	AUG88377.1	Phosphate starvation-inducible protein
ORF 18	6068	6511	147	AUG88378.1	Hypothetical protein
ORF 19	6637	6849	70	AUG88378.1	Hypothetical protein
ORF 20	6865	7806	313	AUG88379.1	Ribonucleotide reductase
ORF 21	7857	9590	577	AUG88380.1	Ribonucleotide reductase
ORF 23	10089	10226	45	AUG88382.1	Hypothetical protein
ORF 24	10357	10545	62	AUG88384.1	Hypothetical protein
ORF 26	10668	11621	317	AUG88385.1	DNA ligase
ORF 28	11758	11904	48	AUG88386.1	Hypothetical protein
ORF 29	11897	12124	75	AUG88387.1	Hypothetical protein
ORF 30	12124	12474	116	AUG88388.1	Hypothetical protein
ORF 31	12446	12742	98	AUG88389.1	Hypothetical protein
ORF 32	12744	13001	85	AUG88390.1	Hypothetical protein
ORF 33	13050	13277	75	AUG88391.1	Hypothetical protein
ORF 34	13312	13542	76	AUG88392.1	Hypothetical protein
ORF 35	13542	14195	217	AUG88393.1	Thymidylate synthase
ORF 38	14636	14788	50	AUG88396.1	Hypothetical protein
ORF 40	14872	15264	130	AUG88397.1	Hypothetical protein
ORF 41	15503	15958	151	AUG88399.1	Hypothetical protein
ORF 42	15945	16217	90	AUG88400.1	Hypothetical protein
ORF 44	16416	17225	269	AUG88401.1	Ribose-phosphate pyrophosphokinase
ORF 45	17218	17394	58	AUG88402.1	Hypothetical protein
ORF 46	17391	17753	120	AUG88403.1	Hypothetical protein
ORF 47	17750	19315	521	AUG88404.1	Nicotinamide phosphoribosyl transferase
ORF 48	19423	19575	50	AUG88406.1	Hypothetical protein
ORF 49	19568	19984	138	AUG88407.1	Hypothetical protein
ORF 50	19974	20408	144	AUG88408.1	Hypothetical protein
ORF 51	20395	20622	75	AUG88410.1	Hypothetical protein
ORF 52	20645	20941	98	AUG88411.1	Hypothetical protein
ORF 53	20944	21411	155	AUG88412.1	Hypothetical protein

ORF 54	21408	21602	64	AUG88413.1	Hypothetical protein
ORF 55	21599	22138	179	AUG88414.1	DNA polymerase
ORF 57	22232	23965	577	AUG88415.1	DNA helicase
ORF 58	24075	24266	63	AUG88416.1	Hypothetical protein
ORF 59	24268	24492	74	AUG88417.1	Hypothetical protein
ORF 60	24489	24725	78	AUG88418.1	Hypothetical protein
ORF 61	24789	24986	65	AUG88419.1	Hypothetical protein
ORF 62	24986	25489	167	AUG88420.1	Hypothetical protein
ORF 64	25736	25942	68	AUG88421.1	Hypothetical protein
ORF 66	26421	26684	87	AUG88423.1	Hypothetical protein
ORF 67	26732	26872	46	AUG88424.1	Hypothetical protein
ORF 68	26875	27078	67	AUG88425.1	Hypothetical protein
ORF 69	27084	27287	67	AUG88426.1	Hypothetical protein
ORF 70	27287	27730	147	AUG88427.1	Hypothetical protein
ORF 71	27911	30235	774	AUG88428.1	Hypothetical protein
ORF 72	30384	30605	73	AUG88430.1	Hypothetical protein
ORF 73	30598	32235	545	AUG88431.1	Hypothetical protein
ORF 74	32250	33344	364	AUG88432.1	Hypothetical protein
ORF 75	33304	33906	200	AUG88433.1	Hypothetical protein
ORF 76	33906	34709	267	AUG88434.1	Hypothetical protein
ORF 77	34711	35583	290	AUG88435.1	Hypothetical protein
ORF 78	35583	35906	107	AUG88436.1	Hypothetical protein
ORF 80	36313	36951	212	AUG88437.1	Hypothetical protein
ORF 81	36970	37281	103	AUG88438.1	Hypothetical protein
ORF 83	37490	37708	72	AUG88439.1	Hypothetical protein
ORF 84	37708	38193	161	AUG88440.1	Hypothetical protein
ORF 89	39763	40050	95	AUG88444.1	Hypothetical protein
ORF 99	42213	42644	143	AUG88445.1	Hypothetical protein
ORF 100	42653	43132	159	AUG88446.1	Hypothetical protein
ORF 104	43744	44094	116	AUG88447.1	Hypothetical protein
ORF 105	44191	44742	183	AUG88448.1	Hypothetical protein
ORF 106	44743	44976	77	AUG88449.1	Hypothetical protein
ORF 107	45190	45708	172	AUG88450.1	Hypothetical protein
ORF 109	46425	46769	114	AUG88451.1	Hypothetical protein
ORF 111	46971	47150	59	QKN88445.1	Hypothetical protein
ORF 112	47229	48185	318	AUG88452.1	Hypothetical protein
ORF 114	48399	49169	256	AUG88453.1	Hypothetical protein
ORF 118	50010	51800	596	AUG88454.1	Hypothetical protein
ORF 119	51816	52499	227	AUG88455.1	Hypothetical protein
ORF 120	52665	53147	160	AUG88456.1	Hypothetical protein
ORF 122	53542	54069	175	AUG88457.1	Hypothetical protein
ORF 126	55288	55899	203	AUG88458.1	Hypothetical protein
ORF 127	55900	56460	186	AUG88459.1	Hypothetical protein
ORF 128	56447	56764	105	AUG88460.1	Hypothetical protein
ORF 129	56972	57319	115	AUG88461.1	Hypothetical protein

ORF 133	58193	58363	56	AFN37266.1	Hypothetical protein
ORF 135	58571	58813	80	AUG88462.1	Hypothetical protein
ORF 138	59074	59292	72	AUG88463.1	Hypothetical protein
ORF 142	60178	60441	87	AUG88464.1	Hypothetical protein
ORF 144	60721	60972	83	AUG88465.1	Hypothetical protein
ORF 146	61151	62134	327	AUG88466.1	Hypothetical protein
ORF 147	62232	62852	206	AUG88467.1	Hypothetical protein
ORF 148	64908	65294	128	AUG88468.1	Hypothetical protein
ORF 149	65294	65710	138	AUG88469.1	Hypothetical protein
ORF 150	65724	66092	122	AUG88470.1	Hypothetical protein
ORF 151	66141	67067	308	AUG88471.1	Hypothetical protein
ORF 152	67127	68650	507	AUG88472.1	Terminase large subunit
ORF 153	68666	70828	720	AUG88473.1	Portal protein
ORF 155	71048	72160	370	AUG88474.1	Hypothetical protein
ORF 156	72293	73222	309	AUG88475.1	Capsid and scaffold protein
ORF 157	73286	74386	366	AUG88476.1	Hypothetical protein
ORF 158	74398	75648	416	AUG88477.1	Hypothetical protein
ORF 159	75660	76016	118	AUG88478.1	Hypothetical protein
ORF 160	76025	76369	114	AUG88479.1	Hypothetical protein
ORF 161	76379	77128	249	AUG88480.1	Hypothetical protein
ORF 162	77138	77980	280	AUG88481.1	Hypothetical protein
ORF 163	77990	80899	969	AUG88482.1	Hypothetical protein
ORF 164	80892	81203	103	AUG88483.1	Hypothetical protein
ORF 165	81336	84230	964	QVJ07756.1	tailprotein
ORF 166	84440	85159	239	AUG88487.1	Hypothetical protein
ORF 167	85172	86968	598	AUG88488.1	Hypothetical protein
ORF 168	86981	89284	767	AUG88489.1	Hypothetical protein
ORF 169	89344	89757	137	AUG88490.1	Hypothetical protein
ORF 170	89757	90725	322	AUG88491.1	Hypothetical protein
ORF 171	90735	91277	180	AUG88492.1	Hypothetical protein
ORF 172	91287	91706	139	AUG88493.1	Hypothetical protein
ORF 173	91693	93300	535	AUG88494.1	Hypothetical protein
ORF 174	93310	99933	2207	AUG88495.1	Hypothetical protein
ORF 175	100043	100489	148	QBX05958.1	VII recombination endonuclease VII
ORF 177	100700	101236	178	AUG88496.1	3'-phosphatase,5'-polynucleotidekinase
ORF 178	101239	101673	144	AUG88497.1	Hypothetical protein
ORF 179	101664	102050	128	AUG88498.1	Hypothetical protein
ORF 181	102204	102971	255	AUG88499.1	Hypothetical protein
ORF 182	103038	103460	140	AUG88500.1	Hypothetical protein
ORF 183	103460	103882	140	AUG88501.1	Hypothetical protein
ORF 185	104035	105114	359	AUG88502.1	exonuclease
ORF 186	105111	105758	215	AUG88503.1	Hypothetical protein
ORF 187	105773	106027	84	AUG88504.1	Hypothetical protein
ORF 188	106027	106200	57	YP 009620899.1	Hypothetical protein
ORF 189	106252	107034	260	AUG88505.1	Hypothetical protein

ORF 191	107612	108088	158	AUG88507.1	Hypothetical protein
ORF 192	108111	108677	188	AUG88508.1	Hypothetical protein
ORF 194	108964	109605	213	AUG88509.1	Hypothetical protein
ORF 195	110029	110196	55	QQG33671.1	
ORF 196	110317	110715	132	AUG88510.1	Hypothetical protein
